# The Effect of Education on Sexual Health of Women with Hypoactive Sexual Desire Disorder: A Randomized Controlled Trial

**Published:** 2014-04

**Authors:** Maasumeh Kaviani, Tahereh Rahnavard, Sara Azima, Masoumeh Emamghoreishi, Nasrin Asadi, Mehrab Sayadi

**Affiliations:** 1 Department of Midwifery, School of Nursing and Midwifery, Shiraz University of Medical Sciences, Shiraz, Iran;; 2 Student Research Committee, Shiraz University of Medical Sciences, Shiraz, Iran;; 3 Community Based Psychiatric Care Research Center, Department of Midwifery, School of Nursing and Midwifery, Shiraz University of Medical Sciences, Shiraz, Iran;; 4 Department of Pharmacology, School of Medicine, Shiraz University of Medical Sciences, Shiraz, Iran;; 5 Department of Gynecology, School of Medicine, Shiraz University of Medical Sciences, Shiraz, Iran;; 6 Department of Biostatistics, School of Public Health, Behbahan University of Medical Sciences, Behbahan, Iran

**Keywords:** Education, Sexual Health, Hypoactive Sexual Desire Disorder

## Abstract

**Background: **Sexuality constitutes an important part of women’s life. Healthy and proper sexual functioning is one of the signs of physical and mental health. The present study aimed to identify the effect of education on sexual health of women with hypoactive sexual desire disorder.

**Methods:** In this randomized clinical trial, 80 married women at reproductive age were randomly divided into a control and an education group. These women participated in this study based on self-reporting of having hypoactive sexual desire disorder. After six weekly educational sessions regarding sexual health**,** percentage of changes in sexual desire was assayed using Hurlbert index of sexual desire. Independent and paired t-test and Chi-square test were used to analyze the data.

**Results:** After the intervention, a significant difference was found between the two groups regarding the sexual desire score (P<0.001). The results also showed a significant difference within groups in this regard (P<0.001).

**Conclusion: **According to the results of this study, it seems that educational intervention regarding sexual health was effective for the women with hypoactive sexual desire disorder. Thus, establishing sexual health education units in different health centers is highly necessary. These centers can help couples to promote their sexual knowledge and treat their sexual dysfunctions.

**Trial Registration Number: **IRCT2012101911032N2

## Introduction

Sexual needs are among the physiological and even spiritual and mystical needs.^1^ Sexuality is beyond sexual activity and is in fact the innermost feelings and deepest aspirations of humans in making a relationship meaningful.^2^ Questions and concerns related to sexuality constitute an important part of women’s life.^3^ In some communities, 80% of women feel that sexual relationship is a necessary component of life.^4^ One of the hallmarks of physical and mental health and a factor of life quality is healthy and proper sexual functioning.^5^

Unawareness about and ignorance of sexual desire cause many psychological distresses and sexual dissatisfaction leads to marital conflicts.^6^ Sexual relationship is the foundation of mental health and survival of a healthy generation. Moreover, any disorder in the bilateral process of communication can lead to emergence of disruption and insecurity in families.^7^ Hypoactive Sexual Desire Disorder (HSDD), from which 1 per 10 women suffer, is one of the main and challenging issues in women’s sexual health and is one of the major reasons for their referral to clinics at all ages.^8^ This disorder has a negative effect on life quality and “feeling of being good”. HSDD not only includes shortage or lack of fantasy or tendency to any form of sexual activity, but also causes personal distress or interpersonal problems for patients. Acquired HSDD occurs after a period of normal sexual desire or tendency and is not related to special conditions, situations, or relationships (for example, change of sexual partner). Negative effects of HSDD on a woman include feeling less feminine, feeling of sexual failure, low self-confidence, lack of security, and feeling inferior in front of a sexual partner. This distress is thus directly related to depression.^9^^-^^12^ In 2004, World Health Organization (WHO) proposed to investigate sexual health independently from reproductive health owing to its importance since many diseases and disorders all over the world are caused by unawareness from sexual health.^13^

Sexual health education is an informed process that forms views and beliefs about sex, sexual identity, and sexual intimacy and includes a broad concept ranging from human sexual anatomy and reproductive health to emotional relationships and reproductive rights. It causes individuals to select their sexual behavior, helps prevent sexually transmitted diseases and sexual abuse, and has a fundamental role in fulfilling sexual needs and enjoying healthy sexuality.

When the education process starts, so does learning. Educating the patients has been accepted as a part of the activities of all staff in healthcare systems.^14^^-^^17^ In terms of educating and as a result of behavior change, public belief is that sexual health education not only has a positive role in prevention of negative outcomes such as sexually transmitted diseases, sexual abuse, and sexual depression, but it also leads to positive outcomes at personal level and interpersonal relationships. These positive results can help couples create proper relationships, enjoy sexual relationship, boost self-confidence and self-esteem, and make informed decisions.^18^ The findings of the studies conducted on the issue have confirmed the effects of these educational programs on sexual disorders.^19^ Also, educated women can be a good source of information transfer to their husbands, children, and peers.^20^ Although studying and identifying sexual behaviors, tendencies, and issues are among the most important reproductive health issues and investigating the problems related to women’s sexual disorders is among the priorities, in eastern cultures, especially Iranian culture, sexual issues are taboo and people rarely discuss sexual subjects in public places or educational institutes. Moreover, in spite of the fact that sexual desire is one of the important issues in fertility health, unfortunately sexual education services centers are rare or people are unaware of existence of these centers. Therefore, the present study aims to assess the effect of sexual health education on fertile women with HSDD.

## Materials and Methods

**Figure 1:**
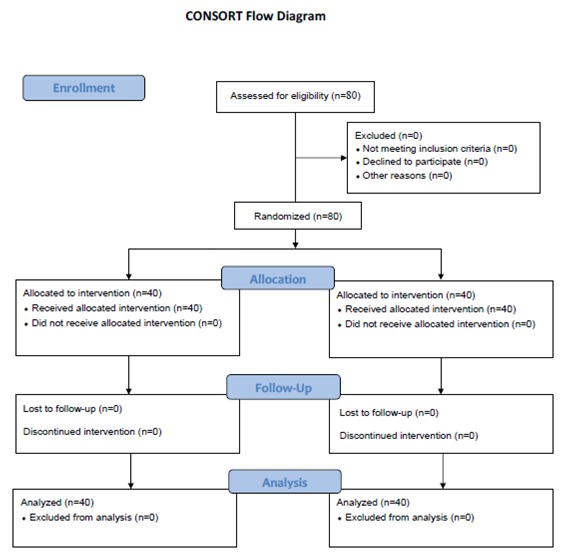
Design and protocol of the study

In this randomized clinical trial, 80 individuals were selected from the women referring to Motahhari women’s clinic, Shiraz, Iran through convenience sampling. The protocol of the study is shown in figure 1.

The women participated in this study based on their self-report of HSDD. While selection of the samples, if one of the patients was not qualified, the next person would be replaced. Afterwards, the samples were divided into two groups using the table of random numbers. Accordingly, numbers 2 and 3 were assigned to group A (sexual health education) and numbers 4 and 5 were assigned to group B (control). It should be mentioned that sampling was done in different days and the two groups never communicated with each other.

Based on the previous studies conducted on the issue and the study objectives, considering error of 5% and power of 80%, and using the following formula, a 70-subject sample size (35 in each group) was determined for the study:

$$n=\frac{{\text{s}}^{2}\left({\text{t}}_{\alpha \text{,}\nu }+{\text{t}}_{\beta \left(1\right)\text{,}\nu }\right)}{{\left(\mathrm{\&part;}\right)}^{2}}$$

According to the longitudinal design of the study and repeated measurements, by using the formula, finally an 80-subject sample size (40 in each group) was determined for the study.

The study data were collected using a questionnaire containing demographic information and sexual indicators.

The inclusion criteria of the study were being married, being 18 to 44 years old, suffering from HSDD, having no internal diseases including cardiovascular diseases, hypothyroidism, arthritis, diabetes, epilepsy, kidney infection, etc., not being pregnant, not breastfeeding, not having emotional-family problems, not consuming oral contraceptive pills, not taking any drugs affecting sexual functioning (such as tricyclic antidepressants, blood pressure lowering drugs, H2blocker anti-histamines, barbiturates, selective serotonin reuptake inhibitors, etc.), not suffering from mental, neurological, or psychological diseases, lack of drug addiction of both the participant and her husband, not being under mental consultation, and not consuming the herbal medications affecting sexual desire. On the other hand, the exclusion criterion of the study was unwillingness to continue participation in the study. In the intervention group, the researcher taught sexual health in six 90-minute sessions. The training was performed through lecture, group participation, watching movies, and question and answer. The educational content included anatomy and physiology of reproductive system in men and women, physiology of sexual response in men and women, Masters W. and Johnson V. sex-specific preparation techniques and relaxation techniques (one session for each), and role of love and sexual relationship, separately and in combination, in sexual relationships for two sessions. The content was prepared from accredited scientific sources.

Demographic information, including age, occupation, education, history of marital life, etc., was collected through interviews before and after the intervention. Besides, sexual relation pattern was evaluated using Hurlbert’s sexual activity questionnaire. This questionnaire was designed in 1992 by Apt and Hurlbert and can be used in both clinical and general population samples. Hurlbert’s sexual activity questionnaire consists of 25 questions scored through a Likert scale ranging from 0 (never) to 4 (always). Thus, the questionnaire’s score ranges from 0 to 100. The lower the score, the weaker the person’s sexual desire and vice versa. The Cronbach’s alpha of this questionnaire was obtained as 0.95 in a foreign study. Additionally, its test-retest reliability was computed as 0.86 in a two-week interval.^21^

The Cronbach’s alpha coefficient of the Persian version of the questionnaire was 0.89 showing acceptable reliability and content validity. In addition, its Kendall’s coefficient was obtained as 0.86.^22^

In this study, the educational classes were held through six 90-minute sessions during 3 days. After six weekly educational sessions, the percentage of changes in sexual desire was assayed again. The intervention was just accomplished for the education group. However, considering ethics, the control group was also educated after the end of the study. They were also able to keep in touch with the researcher and ask their sexual problems. In order to prevent researcher’s partiality and bias, the questionnaires were completed by the participants themselves. Of course, it is clear that the researcher’s presence and explanation for completing the questionnaires was absolutely necessary.

After all, the data were analyzed using descriptive statistics, paired and independent t-test, one-way ANOVA, multiple comparisons, Chi-square test, and nonparametric tests. Besides, P<0.05 was considered as statistically significant. Since equalization of the groups regarding the confounding factors was not possible after randomization, other statistical methods, such as analysis of covariance were used. All the analyses were performed using the SPSS statistical software (version 16).

Ethical Considerations

With the permission of the Research vice-chancellor of Shiraz University of Medical Sciences, Ethics Committee (CT-91-6206), and Iranian Registry of Clinical Trial *(*2012101911032N2*)*, all the women who had the inclusion criteria were interviewed. The objectives and procedures were explained to all the participants and written informed consents for participation in the study were obtained from them.

## Results

This study was conducted on 80 married women (40 in the education group and 40 in the control group).

The study findings revealed no significant difference between the mean age of the women in the intervention group (33.75±6.41) and the control group (34.23±7.62). Also, no significant difference was found between the two groups’ mean age of marriage (22.28±4.99 years in the intervention group and 21.20±3.8 years in the control group). The two groups were also similar regarding the mean duration of marriage (11.43±8.17 years in the intervention group and 13.17±9.64 years in the control group).

Paired *t* test was used to compare each group’s mean scores of Hurlbert’s sexual desire questionnaire before and after the intervention. Paired t-test was also used to compare the two group’s mean scores of Hurlbert’s sexual desire questionnaire before and after the intervention. According to the results, a significant difference was found in the education group’s mean score of Hurlbert’s sexual desire questionnaire after the intervention (P<0.001) (table 1). A significant difference was also observed in the control group in this regard after the intervention (P<0.001) (table 1). Moreover, the results demonstrated a significant difference between the two groups regarding the mean sexual desire scores after the intervention (P<0.001) (table 1). Of course, this difference was also significant before the intervention (P<0.002) (table 1). Thus, the mean of change percentage was compared in the two groups. Accordingly, the mean score of sexual desire index in the sexual health education group was higher compared to the control group (P<0.02) (table 1).

**Table 1:** Comparison of the two groups’ mean scores of Hurlbert’s sexual desire index before and after the intervention

**Sexual** **desire score**	**Groups**		** P value^a^**
	**Sexual health** **education** **mean** **±** **SD**	**Control** **mean** **±** **SD**	
Before the intervention	37.12±15.88	24.77±11.92	0.002
After the intervention	53.35±15.49	32.32±11.86	<0.001
Percentage of changes	75.48±67.43	43.8±42.7	0.02
P value^b^	<0.001	<0.001	-

a: P value, independent *t* test was used; b: Paired *t* test was used to compare the two groups’ scores before and after the intervention

## Discussion

The complex nature of female sexual function requires a holistic treatment approach. In this respect, a range of psychological therapies may be helpful, including basic psychosexual counseling, cognitive behavioral therapy, relationship counseling, and body awareness education.^23^

Nevertheless, limited information is available regarding the efficacy of many of these interventions.^24^

The purpose of this study was to investigate the effect of sexual health education on changes of sexual desire in women with reduced sexual desire. The results showed a significant difference in the intervention group’s sexual desire index after the intervention. Similar results were also obtained in the control group. Moreover, a significant difference was found between the two groups regarding their sexual desire mean scores before and after the intervention. To control sexual desire score due to significance of the scores before the intervention, percentage of change was calculated indicating a significant difference between the two groups before and after the intervention. Accordingly, the mean percentage of changes was higher in the sexual health education group in comparison to the control group. This indicated the effectiveness of the educational intervention in this group, which was in agreement with the results of another study conducted on the issue.^25^ Since one of the major causes of sexual dysfunction is communicational and distress problems in marital relationships, the quality of sexual relationship between couples is one of the most important factors that affect libido, and women’s sexual function is affected by HSDD, use of regular opportunities for sexual health education is important in different stages of life.^26^^,^^27^

Many researches have demonstrated that cognitive-behavioral education is effective in treatment of any mental and physical disorder, even if other treatments also exist for these diseases.^28^

Since one of the components of cognitive-behavioral therapy is proposing sexual information and knowledge, a number of investigators have focused on this issue. For example, some researchers have reported a relationship between sexual abnormality and knowledge.^29^

In this study, Hurlbert’s score was low in both the intervention and the control group. Considering the higher percentage of changes in the sexual health education group, the significant effect of sexual health education can be perceived. Another study also came to the conclusion that when the target group had a low level of knowledge about sexual function, every educational intervention could considerably increase the group members’ awareness and knowledge. Increased awareness could be probably due to acquisition of positive attitude toward gaining appropriate sexual function. The sexual health education group in this study, similar to another study, was aware of the importance of sexual relationships in the marital life and had positive attitude toward enjoying proper sexual relations. However, wrong functioning of the educating group before the intervention for obtaining enjoyable relations was due to their unawareness of the nature of these relations and their lack of access to good resources.^30^

Specific problems in bilateral sexual relations could also be attributed to inadequate sexual information, inappropriate verbal communication of couples, and their poor sexual fantasies. Such couples’ sexual fantasies rarely correspond with each other. In this case, even if the couples have good emotional connection, their sexual relation may face difficulty. Moreover, although some couples have sufficient adaptability and flexibility to start an adequate relationship, they face sexual difficulties solely because of incomplete sexual information or misunderstandings. Such a simple ignorance could lead to severe sexual dysfunction in case there is no verbal communication and proper sexual information.^31^

In one other study, cognitive-behavioral training was used to improve sexual dysfunction. In these studies, similar to the present research, the significant positive effect of education was well recognized.^32^^,^^33^

One study indicated that sexual health education played an important role in family health, positive attitude toward sexual relations, sexual pleasure, reduction of family conflicts, and obtaining sexually pleasurable experiences.^34^ Also, a large number of researches have shown an urgent need for education in the field of sexual health.^30^ Furthermore, some researchers have referred to the theoretical and practical importance of sexual attitude and have recommended to health providers to incorporate change in incompatible beliefs and wrong attitudes in their training. Besides, the significant correlation between sexual knowledge and sexual attitude confirms that as the couple’s knowledge of sexual issues increases, their attitude becomes more flexible.^35^

According to the results presented in table 1, the improvement rate was lower in the women with decreased sexual desire in the control group compared to those in the sexual health education group. The control group’s responses primarily depended on the patients’ medical, cultural, and environmental conditions.^36^ Although little information is available about response to treatment of sexual disorders in control groups,^37^ even this amount of change can reflect its positive impact on women’s sexual desire. This result was similar to that of another study indicating that most clinical trials on women’s sexual disorders have revealed considerable clinical responses in the control group.^38^

Although efforts have been made to separate the treatment results in the intervention and control groups for showing real treatment responses, in practice, it is difficult to separate these responses from real treatment responses.^39^

It is thought that a change in the patients’ behavior is a potential mediating factor in response of the control group^40^^,^^41^ and has the highest relationship with treatment of sexual disorders. Thus, based on the patients’ desires, demands, and expectations, sexual response may increase by change in the patients’ behavior.^34^ Moreover, the findings of the current study revealed some changes in the control group which could be due to the way the researcher affected their mentality, process of completing the questionnaires, or talking about their sexual problems.

In the Iranian society, sexual issues are taboo and this turned to a limitation in sampling and data collection stages. Initially, there were problems in convincing the participants. However, these problems were later eliminated by explaining the research objectives to the participants and establishing proper relationships to gain their trust. 

## Conclusion

Given the results of this study, it is clear that sexual health education is one of the key components of treatment of sexual dysfunction in women with reduced sexual desire.

However, considering the undeniable influence of sexual relations on the quality of family life, it is recommended to hold advanced training workshops in health centers to improve sexual relations and prevent sexual problems as well as marital conflicts so as to proceed toward empowerment of women and promotion of their and their families’ health. 
